# Effectiveness of Organized Mammography Screening for Different Breast Cancer Molecular Subtypes

**DOI:** 10.3390/cancers14194831

**Published:** 2022-10-03

**Authors:** Lilu Ding, Marcel J. W. Greuter, Inge Truyen, Mathijs Goossens, Bert Van der Vegt, Harlinde De Schutter, Guido Van Hal, Geertruida H. de Bock

**Affiliations:** 1 Department of Epidemiology, University Medical Center Groningen, University of Groningen, 9713 GZ Groningen, The Netherlands; 2 Department of Social Epidemiology and Health Policy, University of Antwerp, Antwerp, 2610 Antwerpen, Belgium; 3 Department of Radiology, University Medical Center Groningen, University of Groningen, 9713 GZ Groningen, The Netherlands; 4 Department of Robotics and Mechatronics, University of Twente, 7522 NH Enschede, The Netherlands; 5 Belgian Cancer Registry, Rue Royale 215, 1210 Brussels, Belgium; 6 Center for Cancer Detection (CvKO), Flanders, 8000 Bruges, Belgium; 7 Vrije Universiteit Brussel, 1090 Brussels, Belgium; 8 Department of Pathology & Medical Biology, University Medical Center Groningen, University of Groningen, 9713 GZ Groningen, The Netherlands

**Keywords:** breast neoplasms, early detection of cancer, immunohistochemistry, biomarkers, social participation

## Abstract

**Simple Summary:**

We evaluated the short-term effectiveness of a mammography screening program in all women who participated in the screening program and were diagnosed with screen-detected or interval breast cancer (BC) in Flanders (2008–2018). The evaluation was performed for the major molecular subtypes of invasive BC separately and considering the regularity of participation. We found that screen-detected BC was more likely to be diagnosed at early stages than interval BC of luminal, luminal-HER2-positive, and triple-negative BC (TNBC) type, but not for the human epidermal growth factor receptor 2-positive (HER2 positive) subtype. In addition, regular participation was related to a higher likelihood of screening detection than irregular participation for luminal, luminal-HER2-positive, and TNBC, but not for the HER2 positive subtype, either. Our results indicate that regular screening as compared to irregular screening is effective for all breast cancers except for the HER2 subtype.

**Abstract:**

Background: Screening program effectiveness is generally evaluated for breast cancer (BC) as one disease and without considering the regularity of participation, while this might have an impact on detection rate. Objectives: To evaluate the short-term effectiveness of a mammography screening program for the major molecular subtypes of invasive BC. Methods: All women who participated in the screening program and were diagnosed with screen-detected or interval BC in Flanders were included in the study (2008–2018). Molecular subtypes considered were luminal and luminal-HER2-positive, human epidermal growth factor receptor 2-positive, and triple-negative BC (TNBC). The relationship between the BC stage at diagnosis (early (I–II) versus advanced (III–IV)) and the method of detection (screen-detected or interval) and the relationship between the method of detection and participation regularity (regular versus irregular) were evaluated by multi-variable logistic regression models. All models were performed for each molecular subtype and adjusted for age. Results: Among the 12,318 included women, BC of luminal and luminal-HER2-positive subtypes accounted for 70.9% and 11.3%, respectively. Screen-detected BC was more likely to be diagnosed at early stages than interval BC with varied effect sizes for luminal, luminal-HER2-positive, and TNBC with OR:2.82 (95% CI: 2.45–3.25), OR:2.39 (95% CI: 1.77–3.24), and OR:2.29 (95% CI: 1.34–4.05), respectively. Regular participation was related to a higher likelihood of screening detection than irregular participation for luminal, luminal-HER2-positive, and TNBC with OR:1.21 (95% CI: 1.09–1.34), OR: 1.79 (95% CI: 1.38–2.33), and OR: 1.62 (95% CI: 1.10–2.41), respectively. Conclusions: Regular screening as compared to irregular screening is effective for all breast cancers except for the HER2 subtype.

## 1. Introduction

Invasive breast cancer is a common heterogeneous disease [1]. The main subtypes are luminal, luminal-HER2-positive, human epidermal growth factor receptor 2 (HER2) positive, and triple-negative breast cancer (TNBC) [2,3]. In 2020, breast cancer accounted for one in four newly diagnosed cancer cases and one in six cancer deaths worldwide in women [4]. Population-based organized mammography screening programs have been implemented widely in high-income countries based on the screening’s proven long-term effect on mortality reduction. In randomized controlled trials (RCTs), screening has the potential to reduce up to 40% of breast cancer mortality for attendees [5,6,7]. However, the evaluation of the long-term effect of screening requires sufficient follow-up time because the effect of screening on breast cancer mortality takes at least 10 years to become evident [8]. 

In parallel, many studies evaluated the short-term effect of screening regarding the stage of cancer at diagnosis and the mode of cancer detection (screen-detected or interval). In short term, a more favorable stage of screen-detected cancer compared to interval cancer indicates the effectiveness of screening because diagnosis at an early stage leads to a more favorable prognosis than at an advanced stage [9]. Strikingly, population-based studies on the evaluation of short-term effectiveness of screening normally considered invasive breast cancer as one disease [10,11]. It is well known that invasive breast cancers of different molecular subtypes can have different growth rates [12,13]. Population-based cohort studies showed that ER-negative/HER2-positive breast cancers are considered more aggressive and more likely to be diagnosed as interval cancer [13,14,15,16]. It is not clear if all women diagnosed with different breast cancer molecular subtype benefit from screening equally. Therefore, to evaluate the effectiveness of breast cancer screening, it is necessary to consider the major molecular subtypes of breast cancer. 

The value of breast cancer screening programs is the detection of breast cancer at an early stage [9]. Compared to screen-detected breast cancers, interval cancers generally have more advanced stages and a poorer prognosis [14]. When evaluating the short-term effectiveness of a breast cancer screening program, the number of visits and regularity of participation need to be taken into account [17]. Indeed, regularity of screening attendance can affect both the mode of detection (screen-detected or interval) and the stage of breast cancer diagnosis [14,16,18]. Few studies have attempted to evaluate the effect of consecutive participation in screening and reported that women who participated in the last two screenings before diagnosis have a lower risk of breast cancer than women who participated in only one or none of the last two screenings before diagnosis [19,20,21]. However, these studies only considered the number of screening rounds women participated in, without considering the effect of the regularity of screening participation. 

This study aimed to evaluate the short-term effectiveness of a population-based organized breast cancer screening program when the role of screening regularity and main molecular breast cancer subtypes are considered. To that goal, we linked the data of screening participation and diagnosed breast cancer molecular subtypes at the individual level. As molecular subtypes are not commonly investigated for in-situ breast cancers, we only included invasive breast cancers for this study.

## 2. Methods

### 2.1. The Inclusion and Exclusion Criteria of the Study Population

This study included all women who were ever screened in the population-based organized breast cancer screening program in Flanders and diagnosed as screen-related invasive BC (diagnosed ≤24 months after screening) from 2008 to 2018. Since the information on breast cancer stage and hormone receptors were only available from 2008 to 2018, we excluded women who had their last screening after 2016 to ensure all women had a complete follow-up time of 24 months after their last screening. In addition, all prevalent screen-detected and interval cancers within 2 years of a prevalent screen were excluded. 

The biennial invitation to the organized breast cancer screening program for all women aged 50 to 69 with no history of breast cancer in Flanders was started in 2001 [22]. The breast cancer screening program was implemented in an organized way in the sense that a dedicated center for cancer detection (CCD) was installed for the organization of the program with systematic quality control measures consistent with the European guidelines [17,22]. No extra exams besides the mammography screening test were provided at the time of screening. Furthermore, at the time of screening, no extra explanation was provided unless women specifically asked for it. 

### 2.2. Data Sources 

The Belgian Cancer Registry received approval (reference number 14/115) from the Belgian Sectoral Committee of Social Security and Health to collect and deterministically link Belgian Cancer Registry (BCR), population-based mammography screening program, and InterMutualistisch Agentschap (IMA) data, using the social security number as a unique patient identifier to evaluate the quality of breast cancer screening in Flanders. The individual-level data on participation in the screening program and the breast cancer diagnoses were linked from the CCD and the BCR, respectively. Specifically, the CCD provided the age, screening date, and screening results of participating women, and the BCR provided the cancer incidence date and the age at the time of diagnosis, and pathological characteristics including stage, and estrogen receptor (ER), progesterone receptor (PR), and human epidermal growth factor receptor 2 (HER2) status. Stages were defined according to the TNM classification system, and pathological stages were prioritized over clinical stages with the exception of distant metastases which were always considered stage IV. Invasive breast cancers that were down staged following neoadjuvant therapy were classified with unknown stages. All data were linked at the individual level using the national social security number as a unique personal identifier. Only pseudonymized data were available to the researchers, within a strictly secured environment in line with the European Union General Data Protection Regulation. Moreover, informed consent from the participants was obtained at the time of screening (Supplementary Materials). All women were informed of the option to opt-out of the use of their data for any research purpose at the time of screening. The rate of activated opt-out in the past years was around 1% of the screened women [22]. 

### 2.3. Outcomes

In this study, the short-term effectiveness of the screening program was primarily characterized by the percentage of early-stage breast cancers and secondary by the percentage of screen-detected breast cancers. Therefore, the primary outcome was the stage of invasive breast cancer at diagnosis categorized into early stage (I, II) and advanced stage (III, IV). The secondary outcome was the breast cancer detection mode: screen-detected breast cancers were defined as diagnosed within 3 months of the first diagnostic assessment that followed a positive screen (but at the latest within 24 months of screening), whereas interval breast cancers were defined as diagnosed within 24 months after a negative screening or diagnosed more than 3 months after the first diagnostic assessment that followed a positive screen (but at the latest within 24 months of screening).

The molecular subtypes were approximated by the joint expression of ER, PR, and HER2 status [2]. All cancers were categorized into five groups: luminal with ER and/or PR positive and HER2 negative; luminal-HER2-positive with ER and/or PR positive and HER2 positive; HER2-positive with ER, PR negative and HER2 positive; the TNBC with ER, PR, and HER2 negative; all other cancers were categorized into the group with unknown molecular type. 

### 2.4. Determinants 

Screening regularity: The regular screening participants were defined as women who had a per woman uptake of screening ≥70% and a per woman averaged screening interval ≥20 months and ≤28 months. Based on a similar idea of the participation rate for the whole population [17], the per women uptake of screening was defined as the number of screenings attended divided by the total number of screening opportunities. For women who were diagnosed before age 69, the endpoint of the calculation of screening opportunity was the cancer diagnosis date. Therefore, the irregular screening participants were defined as women who had a per women uptake of screening <70% and/or had an average screening interval >28 months or <20 months. We applied the 20 to 28 months rather than a fixed 24-month average screening interval in the definition of screening regularity in order to account for the variability in the screening interval in practice. Age at diagnosis: the age of women at the diagnosis of invasive breast cancer was categorized into four age groups, 50–54, 55–59, 60–64, and 65–71. 

### 2.5. Statistical Analysis

For the primary outcome, the likelihood of early-stage diagnosis was compared between screen-detected and interval cancers first with univariate logistic regression models and subsequently with multivariable logistic regression models with adjustment of age at diagnosis and screening participation regularity. Invasive breast cancers with unknown stages were not included in this analysis. For the secondary outcome, the likelihood of diagnosis as screen-detected was compared for regular and irregular screening with multivariable logistic regression models with adjustment of the age at diagnosis. All analyses were performed for breast cancers of different molecular subtypes separately. 

For the primary outcome, we performed a sensitivity analysis to account for the potential impact of overdiagnosis on the stage of a cancer diagnosis. Since overdiagnosis of cancer can be defined as the detection of cancer that would have never become symptomatic if not screened [23]. Thus, overdiagnosis can dilute the proportion of advanced-stage cancers. In the sensitivity analyses, 10% of screen-detected early-stage breast cancer was assumed as overdiagnosed and randomly excluded. As the overdiagnosis rate in the Flanders breast cancer screening program is not reported in the literature, we applied this published data from the Dutch population [23,24]. 

Odds ratio (OR) and corresponding 95% confidence interval (CI) were reported as the effect size from the regression models. All statistical tests were two-sided with a statistical significance level of 0.05. The analyses were performed in R 4.0.5.

## 3. Results

In total 12,318 women were diagnosed with screen-related breast cancer and included in this study, of which luminal was the most commonly diagnosed breast cancer and accounted for 70.9% of the total diagnosed breast cancers followed by luminal-HER2-positive, TNBC, and HER2-positive breast cancers at 11.3%, 4.7%, and 1.8%, respectively (Table 1). The percentage of screen-detected luminal and luminal-HER2-positive breast cancer was 62.9% and 56.1%, respectively, while only less than 50% of TNBC and HER2-positive breast cancer were diagnosed by screening (Table 1). Overall, 87.3% of breast cancers were diagnosed at an early stage (I, II), and only 1.6% (*n* = 203) of all included breast cancers were classified as an unknown stage. More breast cancers were diagnosed at an early stage (I, II) in regularly screened women than in irregularly screened women for overall, luminal, luminal-HER2-positive, and triple-negative breast cancers (Table 1).

Overall, the percentage of screen-detected and interval early-stage breast cancer was 93.0% and 82.2%, respectively. More screen-detected breast cancers were diagnosed at early stages than interval breast cancer for all molecular subtypes. In univariate logistic regression models, the tests were statistically significant overall and for all molecular subtypes except for HER2-positive breast cancer (Table 2). In the multivariable logistic regression model, screen-detected breast cancer was statistically significantly related to a higher likelihood of early stage at diagnosis than interval breast cancer with OR: 2.84 (95%CI: 2.53–3.20). This was also the case for the luminal, luminal-HER2-positive, TNBC, and unknown molecular subtypes with OR: 2.82 (95%CI: 2.45–3.25), OR: 2.39 (95%CI: 1.77–3.24), OR: 2.29 (95%CI: 1.34–4.05) and OR: 3.95 (95%CI: 2.75–5.73), respectively (Table 3). Regular screening was statistically significantly related to a higher likelihood of screen-detection cancers overall with OR: 1.28 (95%CI: 1.18–1.40) and for breast cancer of luminal, luminal-HER2-positive, TNBC, and unknown molecular subtype with OR: 1.21 (95%CI: 1.09–1.34), OR: 1.79 (95%CI: 1.38–2.33), OR: 1.62 (95%CI: 1.10–2.41), and OR: 1.37 (95%CI: 1.05–1.81), respectively (Table 4). 

The sensitivity analysis with a 10% overdiagnosis rate showed that screen-detected breast cancer was statistically significantly related to a higher likelihood of early-stage breast cancer than interval breast cancer. The effect size decreased slightly compared to the results in Table 3 for luminal (OR: 2.54 (95%CI: 2.20–2.93)), luminal-HER2-positive (OR: 2.15 (95%CI: 1.59–2.92)), TNBC (OR: 2.07 (95%CI: 1.21–3.67)), and unknown molecular subtype (OR: 3.56 (95%CI: 2.48–5.16)) (Supplementary Table S1).

## 4. Discussion

### 4.1. Main Findings

In this study, we evaluated the short-term effectiveness of the organized breast cancer screening program with the role of screening regularity and the major molecular subtypes considered. We found that for the most commonly diagnosed luminal breast cancers, more than 60% of the cancers were detected in screening and nearly 90% of the cancers were diagnosed at an early stage, while less than 50% of TNBC and HER2-positive breast cancers were diagnosed by screening. Screen-detected breast cancer was statistically significantly related to a higher likelihood of early stages at diagnosis than interval breast cancer for all the molecular subtypes except for the HER2 positive breast cancer. Regularly screened women were more likely to be diagnosed by screening than irregularly screened women.

### 4.2. Comparison with Literature

Over 70% of breast cancers were diagnosed as luminal and more than 11% of breast cancers were diagnosed as luminal-HER2-positive in our study. Despite a large variety of the reported distribution of molecular subtypes in population-based cohort studies, luminal breast cancer is the dominant type in all studies ranging between 54.8% and 77.6% [1,15,16,25,26,27,28,29]. The percentage of luminal-HER2-positive breast cancer in our study is also comparable with the published studies ranging between 7% and 12.5% [1,15,16,25,26,27,28,29]. In contrast, the HER2 positive breast cancer and the TNBC which account for 1.8% and 4.7%, respectively in our study are less than published data in which the range of HER2 positive breast cancer is between 3.0% and 9.7% and the range of the TNBC is between 7.9% and 12.0% [1,15,16,25,26,27,28,29,30].

As is shown in published data, the low incidence of TNBC is age-related, around 37% of the cases of TNBC are diagnosed in women under the age of 50 [31]. Thus, a possible reason for the lower level of TNBC and HER2 positive breast cancer in our study compared to the published studies is that the published studies include women diagnosed at younger ages before 50, while we focused on a population with the age of breast cancer diagnosis ≥50. In addition, hormone-positive breast cancers (luminal and luminal-HER2-positive) have a later onset peak and hormone-negative breast cancers (HER2-positive and TNBC) have an earlier onset peak [29]. The studied population in our cohort has an older age at diagnosis and is, therefore, more likely to include more luminal and luminal-HER2-positive breast cancers and fewer TNBC and HER2-positive breast cancers. The low number of TNBC and HER2-positive breast cancer in our study might also be related to a lower diagnostic rate of regular screening. These high proliferative breast cancers are more likely to be missed in regular screening. Since we performed the analyses for breast cancer of different molecular subtypes separately, the selection will not affect the evaluation of the effectiveness of screening. 

We found that the luminal and luminal-HER2-positive breast cancers are more likely to be detected in screening than the TNBC and the HER2 positive breast cancers which is similar as reported in registry-based cohort studies with women screened between 40 and 70 in Asian, European and North American countries [13,14,15,32]. 

In our study, the screen-detected breast cancers had more favorable stage than interval cancers. Similar results are also reported in published studies and clearly indicate the short-term effect of screening. In addition to these results, we further found that the screen-detected luminal and luminal-HER2-positive breast cancers were more likely to be early stage than the TNBC. For the HER2 positive breast cancers, screening is not effective. This observation is new and has never been reported in published studies which normally evaluated the effectiveness of screening for breast cancer as one disease. Furthermore, we also found that regularly screened women were statistically significantly more likely to be diagnosed in screening than irregularly screened women which is not previously reported. 

### 4.3. Strengths and Limitations

The strength of this study is that the included breast cancers were identified from a large population with more than a decade of follow-up time. The population-based screening participation data and breast cancer diagnosis data were linked at an individual level.

The study also has limitations. First, the molecular subtypes of the diagnosed breast cancers were approximated by the combination of ER, PR, and HER2 status. Breast cancer can be classified into more detailed groups with data like Ki67 [30,33]. For example, there is an increasing number of studies showing that TNBC is also a heterogeneous disease [30]. For this study, we did not have data for such further classification. Nevertheless, based on the current data, we did observe the different likelihood of early-stage breast cancer at diagnosis between screen-detected and interval breast cancer when the comparison was made for breast cancer of different molecular subtypes. Second, the molecular type of around 10% of the breast cancers in our study was unknown. Even so, the overall percentage of unknown molecular types is comparable with data reported in a study that used data from population-based cohorts in which breast cancers of unknown molecular types range between 7% and 20% [27,28]. Third, overdiagnosis is an inevitable unwanted result of screening, we do not know the exact level of overdiagnosis in our included population. We have tried to evaluate the impact of overdiagnosis on our results with sensitivity analysis and found that screen-detected breast cancers remained statistically significantly related to early-stage breast cancer at diagnosis in most breast cancer subtypes. Lastly, we did not obtain long-term follow-up data on the diagnosed breast cancers in different molecular subtypes. As is shown in published studies, although early-stage breast cancer, no matter the subtype, has excellent 5-year distant relapse-free survival without chemotherapy [34], the prognosis of breast cancer of different molecular subtypes diverged in long follow-up time [35,36]. For example, ER- tumors appear to be less prone to death than ER+ tumors in a follow-up time longer than eight years after diagnosis [37] and ER+ tumors have a significantally high level of recurrence in long-term follow-up [35]. Future studies with long-term follow-up time are needed to evaluate the role of earlier diagnosis and the policy for adjuvant treatment and outcome. 

### 4.4. Interpretation of the Findings

Breast cancer is commonly reported in the literature as one disease. However, it represents a spectrum of tumors with heterogeneous growth rates ranging from indolent to aggressive [33]. The most commonly diagnosed luminal and luminal-HER2-positive breast cancers are generally slow-growing [30,38]. In contrast, the TNBC and HER2positive breast cancers are more aggressive and have shorter tumor volume doubling times [30,38] which leads to a shorter time window for screening to detect the tumors. In our results, we found the likelihood of early-stage diagnosis was significantly higher in screen-detected breast cancer than in interval breast cancer. In addition, the odds ratio of early stage at diagnosis varied for breast cancer of different molecular subtypes, and for HER2-positive breast cancer, the difference was not significant. This verifies the heterogeneous nature of the diagnosed breast cancers and the necessity to evaluate the screening effectiveness with the major molecular subtypes of breast cancer considered. 

In most high-income countries, the selection of women eligible for screening is unanimously based on the age of women and the biennial screening interval has been applied for decades [5,39]. The screening frequency has to be coherent with the tumor’s natural history in order to have most of the cancers diagnosed earlier in screening, especially for the more aggressive tumors [14]. Besides the suitable frequency of screening, an adequate number of screening exposures is also necessary. The European guideline for quality assurance in breast cancer screening and diagnosis recommends that 70% of women invited to screening need to bescreened [17]. In our study, we took this quality indicator into account and further refined it by taking the interval between consecutive screenings into account and by defining the regularity of screening. We found that for breast cancer of luminal, luminal-HER2-positive, and TNBC subtypes, which accounted for 86.8% of the subtypes, regularly screened women had a statistically significant higher likelihood of being detected by screening than the irregularly screened women. Our results suggest that organized breast cancer screening is effective for the majority of women eligible for screening and regular participation is key to achieving an effective screening. 

For future studies, an interesting point for investigation is the characteristics of previous screening mammographies before breast cancer diagnosis. If radiology imaging markers of the aggressive TNBC and HER 2 positive breast cancers can be identified on the mammograms, intervention could be developed and implemented in an early phase. Meanwhile, the cost-effectiveness of breast cancer screening is also an important point to consider for breast cancer of different molecular types. Especially for higher proliferative cases, the cost-effectiveness of personalized screening should be evaluated before actual implementation.

## 5. Conclusions

Screen-detected breast cancer was related to a higher likelihood of early-stage breast cancer at diagnosis for all molecular subtypes except for HER2-positive breast cancer. Regular participation in organized breast cancer screening programs was related to more screen-detected breast cancers for the luminal, luminal-HER2-positive, and TNBC subtypes accounting for 86.8% of all diagnosed breast cancers. Women should be informed of the benefit of regular screening participation, encouraging them to participate regularly. Prediction models are needed to identify women at a higher risk of proliferation to facilitate a more personalized screening scheme in the future.

## Figures and Tables

**Table 1:** The number, % screen detected, % early stage of diagnosed breast cancers, in total and per molecular subtype, overall, and stratified by regular screenings behavior and age category (*n* = 12,318).

	Overall	Regular Screening Behavior		Age Category at Breast Cancer Diagnosis			
		Yes	No	50–54	55–59	60–64	65–71
**All subtypes combined**							
Total *N*	12,318	3757	8561	1464	3272	3410	4172
Screen detected %	61.0%	65.7%	58.9%	56.7%	59.6%	62.2%	64.2%
Early stage (I, II) % *	87.3%	88.8%	86.7%	87.1%	87.2%	87.3%	87.5%
**Luminal**							
Subtotal *N* (%)	8739 (70.9%)	2741	5998	1033	2274	2450	2982
Screen detected %	62.9%	66.6%	61.2%	59.2%	61.2%	64.5%	64.2%
Early stage (I, II) %	88.7%	89.8%	88.3%	89.4%	88.9%	88.0%	89.0%
**Luminal-HER2-positive**							
Subtotal *N* (%)	1386 (11.3%)	417	969	165	420	372	429
Screen detected %	56.1%	66.7%	51.5%	50.3%	53.1%	56.5%	60.8%
Early stage (I, II) %	81.8%	87.5%	79.4%	75.8%	80.5%	85.5%	82.3%
**HER2 positive**							
Subtotal *N* (%)	216 (1.8%)	65	150	29	52	54	81
Screen detected %	42.6%	36.9%	45.3%	51.7%	57.7%	35.2%	34.6%
Early stage (I, II) %	80.6%	76.9%	82.7%	75.9%	90.4%	87.0%	71.6%
**TNBC**							
Subtotal *N* (%)	573 (4.7%)	175	398	81	146	153	193
Screen detected %	44.3%	53.1%	40.5%	37.0%	44.5%	48.4%	44.0%
Early stage (I, II) %	86.4%	87.4%	85.9%	81.5%	85.6%	89.5%	86.5%
**Unknown molecular type**							
Subtotal *N* (%)	1342 (11.4%)	345	997	148	361	363	470
Screen detected %	64.1%	69.6%	62.2%	60.1%	63.7%	64.2%	65.5%
Early stage (I, II) %	89.3%	87.8%	88.6%	91.9%	87.3%	87.6%	88.7%

* percentages of early stage calculated on the total cases with the 203 unknown stage cases included.

**Table 2:** Univariate logistic regression model for the comparison of the likelihood of early-stage breast cancer at diagnosis between screen-detected and interval breast cancer. (*n* = 12,115).

	Early Stage	Advanced Stage	OR (95%CI)
Total *			
Interval	3864 (82.2%)	836 (17.8%)	ref
Screen-detected	6893 (93.0%)	522 (7.0%)	**2.86 (2.54–3.21)**
**Luminal**			
Interval	2664 (83.4%)	532 (16.6%)	ref
Screen-detected	5091 (93.4%)	360 (6.6%)	**2.82 (2.45–3.26)**
**Luminal-HER2-positive**			
Interval	454 (76.9%)	136 (23.1%)	ref
Screen-detected	680 (89.2%)	82 (10.8%)	**2.48 (1.85–3.36)**
**HER2 positive**			
Interval	95 (79.8%)	24 (20.2%)	ref
Screen-detected	79 (88.8%)	10 (11.2%)	2.00 (0.92–4.60)
**TNBC**			
Interval	262 (83.7%)	51 (16.3%)	ref
Screen-detected	233 (92.1%)	20 (7.9%)	**2.27 (1.33–4.00)**
**Unknown molecular type**			
Interval	389 (80.7%)	93 (19.3%)	ref
Screen-detected	810 (94.2%)	50 (5.8%)	**3.87 (2.70–5.61)**

* The 203 breast cancers with unknown stage were not included, which accounted for 1.6% of the total included breast cancers.

**Table 3:** Multivariable model for the comparison of the likelihood of early-stage breast cancer at diagnosis for screen-detected and interval breast cancers (*n* = 12,115).

Variable	OR (95%CI)					
	All	Luminal	Luminal-HER2- Positive	HER2 Positive	TNBC	Unknown Molecular Type
**Mode of detection**						
Interval	ref	ref	ref	ref	ref	ref
Screen-detected	**2.84 (2.53–3.20)**	**2.82 (2.45–3.25)**	**2.39 (1.77–3.24)**	1.79 (0.80–4.24)	**2.29 (1.34–4.05)**	**3.95 (2.75–5.73)**
**Age at breast cancer diagnosis**						
50–54	ref	ref	ref	ref	ref	ref
55–59	0.95 (0.78–1.16)	0.92 (0.71–1.18)	1.19 (0.73–1.90)	2.78 (0.67–12.38)	1.21 (0.55–2.59)	0.56 (0.26–1.13)
60–64	0.89 (0.72–1.09)	0.78 (0.61–1.00)	1.38 (0.83–2.29)	2.78 (0.67–11.70)	1.75 (0.75–4.06)	0.50 (0.23–1.00)
65–71	0.92 (0.75–1.12)	0.88 (0.68–1.13)	1.05 (0.63–1.72)	0.93 (0.25–3.10)	1.63 (0.71–3.67)	0.57 (0.26–1.14)
**Screening regularity**						
irregular	ref	ref	ref	ref	ref	ref
regular	1.15 (1.00–1.32)	1.20 (1.01–1.42)	1.48 (1.02–2.17)	0.67 (0.28–1.62)	0.75 (0.41–1.40)	0.92 (0.61–1.40)

**Table 4:** The effect of screening regularity on the model of breast cancer detection (screen-detected vs. interval) (*n* = 12,115).

Molecular Type	Regular Attenders vs. Irregular Attenders OR (95%CI)	
	Crude	Age-Adjusted
Luminal A	**1.26 (1.14–1.38)**	**1.21 (1.09–1.34)**
Luminal-HER2-positive	**1.85 (1.46–2.36)**	**1.79 (1.38–2.33)**
HER2 positive	0.64 (0.35–1.16)	0.95 (0.48–1.89)
TNBC	**1.64 (1.14–2.35)**	**1.62 (1.10–2.41)**
Unknown molecular type	**1.39 (1.07–1.81)**	**1.37 (1.05–1.81)**
Total	**1.32 (1.22–1.43)**	**1.28 (1.18–1.40)**

## Data Availability

Access to the data is possible with approval from the InterMutualistic Agency, the Belgian Cancer Registry, and the Center for Cancer Detection in Flanders. Further information is available from the corresponding author upon request.

## Supplementary Materials

The following supporting information can be downloaded at: https://www.mdpi.com/article/10.3390/cancers14194831/s1, Table S1: Multivariable model for the comparison of the likelihood of early-stage breast cancer at diagnosis for screen-detected and interval breast cancer in sensitivity analyses with 10% assumption of overdiagnosis rate.
