# Supplementary material for: Effectiveness of Organized Mammography Screening for Different Breast Cancer Molecular Subtypes
Source: Cancers (Basel). 2022 Oct 3;14(19):4831. doi: 10.3390/cancers14194831 (PMC9562677; doi:10.3390/cancers14194831)
Supplement: Supplementary file 1 [file cancers-14-04831-s001.zip › Table S1.pdf]

Table S1. Multivariable model for the comparison of the likelihood of early-stage breast cancer at diagnosis for screen-detected and interval breast cancer in sensitivity analyses with 10% assumption of overdiagnosis rate.

Variable	OR (95%CI)					
	**All**	**Luminal A**	**Luminal B**	**HER2 positive**	**TNBC**	**Unknown molecular type**
**Mode of detection**						
Interval	ref	ref	ref	ref	ref	ref
Screen-detected	**2.56 (2.28–2.88)**	**2.54 (2.20–2.93)**	**2.15 (1.59–2.92)**	1.60 (0.71–3.83)	**2.07 (1.21–3.67)**	**3.56 (2.48–5.16)**
**Age at breast cancer diagnosis**						
50–54	ref	ref	ref	ref	ref	ref
55–59	0.95 (0.78–1.16)	0.92 (0.72–1.18)	1.16 (0.71–1.85)	2.68 (0.64–11.90)	1.20 (0.54–2.58)	0.56 (0.26–1.13)
60–64	0.89 (0.73–1.09)	0.78 (0.61–1.00)	1.39 (0.83–2.30)	2.57 (0.62–10.85)	1.70 (0.73–3.96)	0.50 (0.23–1.01)
65–71	0.91 (0.74–1.11)	0.88 (0.69–1.13)	1.03 (0.62–1.69)	0.86 (0.23–2.86)	1.58 (0.69–3.56)	0.57 (0.26–1.13)
**Screening regularity**						
irregular	ref	ref	ref	ref	ref	ref
regular	1.15 (1.00–1.32)	1.20 (1.01–1.42)	1.47 (1.02–2.16)	0.70 (0.29–1.68)	0.76 (0.41–1.41)	0.90 (0.60–1.38)
